# The targeting of AKR1C1 synergizes with gefitinib via the STAT3 signaling pathway in *EGFR*-mutated NSCLC

**DOI:** 10.1016/j.gendis.2025.101633

**Published:** 2025-04-10

**Authors:** Linlin Chang, Shuzhen Wei, Xiaotian Qi, Yang Gao, Jinhua Chen, Yu Cui, Pengxing He, Wenzhou Zhang

**Affiliations:** a Department of Pharmacy, The Affiliated Cancer Hospital of Zhengzhou University & Henan Cancer Hospital, Henan Engineering Research Center for Tumour Precision Medicine and Comprehensive Evaluation, Henan Cancer Hospital, Henan Provincial Key Laboratory of Anticancer Drug Research, Zhengzhou, Henan 450008, China; b School of Pharmaceutical Sciences, Zhengzhou University, Zhengzhou, Henan 450001, China; c Key Laboratory of Immune Microenvironment and Inflammatory Disease Research in Universities of Shandong Province, School of Basic Medical Sciences, Shandong Second Medical University, Weifang, Shandong 261053, China; d Department of Hematopathy, Henan Institute of Hematology, The Affiliated Cancer Hospital of Zhengzhou University & Henan Cancer Hospital, Zhengzhou, Henan 450008, China

As the first-generation tyrosine kinase inhibitor for epidermal growth factor receptor (EGFR-TKI), gefitinib has been proven effective for patients with Del19 or L858R mutations in *EGFR*. Secondary mutations in *EGFR* (mainly T790M) confer resistance to gefitinib in ∼50% of patients.^1^ However, resistance still occurs in other patients without secondary mutations in EGFR, which indicates that EGFR-independent mechanisms also play a vital role in the gefitinib resistance cascade, which deserves further investigation. Our previous studies have demonstrated that aldo-keto reductase 1C1 (AKR1C1) promotes non-small cell lung cancer (NSCLC) metastasis by directly interacting with signal transducer and activator of transcription-3 (STAT3) and reinforcing its activity.^2^ Although studies have focused on the protumor roles of AKR1C1, the role of AKR1C1 in gefitinib resistance is still unknown.

In our previous enriched resistance model, the T4 subpopulation was resistant to several drugs, including the first-generation EGFR-TKIs gefitinib and erlotinib.^3^ Further microarray analysis revealed that up-regulation of *AKR1C1/2* expression was the second highest in T4 cells (Fig. S1A). Overall survival analysis of lung cancer patients receiving pharmaceutical therapy revealed that the *AKR1C1* high group presented a greater hazard ratio versus the *AKR1C1* low group (Fig. S1B, C). These data suggest the potential role of AKR1C1 in EGFR-TKI resistance (Fig. 1A). Furthermore, the AKR1C1 protein level was increased in PC-9 GEF cells and tumors, as indicated by immunohistochemistry staining and western blotting (Fig. S1D–F). However, an increase in AKR1C1 protein was not identified in PC-9 OR cells, which are resistant to the third-generation EGFR-TKI osimertinib (Fig. S1F).

**Figure 1:**
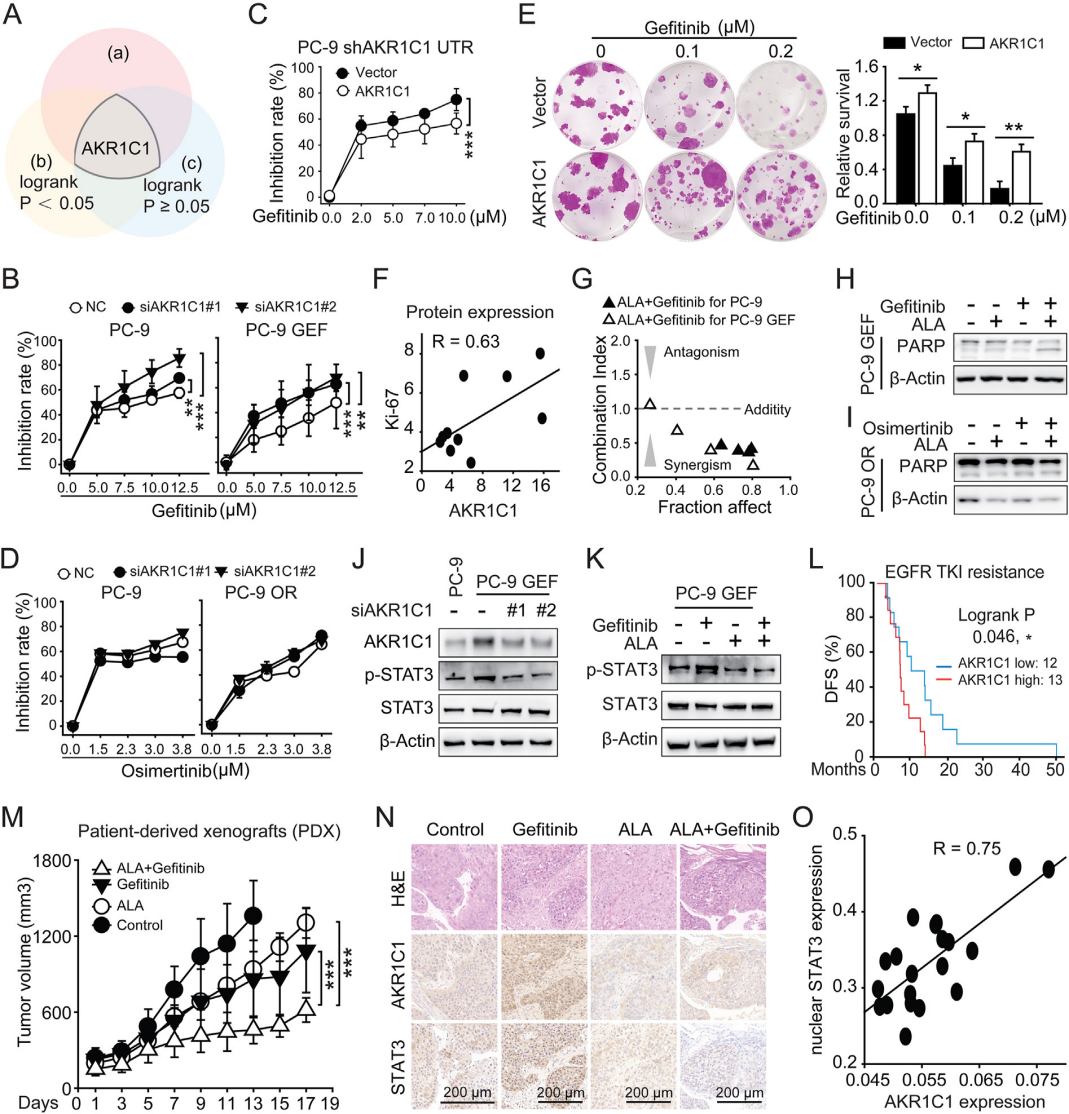
The targeting of AKR1C1 synergizes with gefitinib via the STAT3 signaling pathway in *EGFR*-mutated non-small cell lung cancer (NSCLC). **(A)** The Venn diagrams showing the possible role of AKR1C1 in EGFR-TKI resistance in NSCLC. (a) Heatmap of the expression levels of the top genes in the previous TKI resistance model. (b) The hazard ratio (HR) for the overall survival of lung cancer patients receiving pharmaceutical therapy was obtained from the Kaplan–Meier plotter database. (c) The HR for overall survival of lung cancer patients who did not receive pharmaceutical therapy was obtained from the Kaplan–Meier plotter database. **(B)** AKR1C1 knockdown sensitized both the PC-9 and PC-9 GEF cell lines to gefitinib. **(C)** CCK8 assays were conducted to evaluate the inhibition rates of the AKR1C1 groups and the Vector groups with gefitinib treatments in the PC-9 shAKR1C1 cell line. **(D)** AKR1C1 knockdown had a minor effect on the therapeutic efficacy of osimertinib in both the PC-9 and PC-9 OR cell lines, as shown by the CCK8 assay. **(E)** Cloning assays followed by SRB staining were conducted to evaluate the long-term effects of gefitinib on HCC827 cells stably overexpressing AKR1C1. **(F)** Correlation analysis was performed between the AKR1C1 protein and the Ki67 protein in PC-9 GEF xenograft tumors, both with and without gefitinib treatment. **(G)** Synergistic effects of the targeting of AKR1C1 in combination with gefitinib are shown by the combination index (CI) in PC-9 (black triangle) or PC-9 GEF (white triangle) cells. **(H)** Western blotting was used to investigate the protein expression levels of PARP in the different groups. PC-9 GEF cells were treated with 5.0 μM ALA, 5.0 μM gefitinib, or their combination for two days. **(I)** PARP expression was measured by western blotting in PC-9 OR cells subjected to different treatments. **(J)** Western blotting was conducted to evaluate the protein levels of p-STAT3, STAT3, and AKR1C1 in PC-9 and PC-9 GEF cells after AKR1C1 knockdown. **(K)** The AKR1C1 inhibitor ALA (5 μM) impeded p-STAT3 activation induced by gefitinib (5 μM) in PC-9 GEF cells at 24 h. **(L)** In the OncoSG database, survival analysis was performed using NSCLC patients with EGFR-mutated tumors or with EGFR-TKI resistance. **(M)** The tumor volume curves reflect the effects of combining ALA and gefitinib in a patient-derived xenograft (PDX) model. **(N)** Representative images from the immunohistochemical assay showing the hematoxylin-eosin staining and the staining of AKR1C1 and STAT3 from the PDX model. **(O)** Each plot represents the expression of AKR1C1 and nuclear STAT3 in a separate PDX tumor. Nuclear STAT3 and AKR1C1 staining were quantified by Aipathwell software. The correlation coefficient value (R) was calculated by the CORREL function in Excel software. 0.5 < *R* < 1.0, a highly positive correlation. *P*-values ≥0.05 indicate no significance. ∗*P* < 0.05, ∗∗*P* < 0.01, and ∗∗∗*P* < 0.001.

To explore the role of AKR1C1 in gefitinib resistance, we manipulated the expression of AKR1C1. In PC-9 and PC-9 GEF cells, AKR1C1 knockdown significantly increased the inhibition ratio of gefitinib in a concentration-dependent manner (versus NC; Fig. 1B; Fig. S2A). Furthermore, AKR1C1 overexpression restored resistance to gefitinib in PC-9 cells after AKR1C1 knockdown (Fig. 1C; Fig. S2B). Similar results were also observed in HCC827 cells (Fig. S2C, D). However, in PC-9 and PC-9 OR cells, significant inhibition ratios of osimertinib were not observed after AKR1C1 knockdown (Fig. 1D; Fig. S2E). These data reveal an important role of AKR1C1 in gefitinib resistance but a less important role of AKR1C1 in osimertinib resistance. For long-term effects of AKR1C1 upon gefitinib treatment, a colony formation assay revealed that AKR1C1 conferred a growth advantage to HCC827 cells at each concentration (Fig. 1E). The immunohistochemistry staining results revealed a moderate positive correlation between the AKR1C1 and Ki67 proteins, indicating that gefitinib was not as effective in treating PC-9 GEF tumors (Fig. S2F; Fig. 1F). These results provide additional evidence that AKR1C1 contributes to gefitinib treatment relapse and controls the therapeutic efficacy of gefitinib in *EGFR*-mutated cells both *in vitro* and *in vivo*.

We subsequently introduced an AKR1C1 inhibitor, ALA,^4^ to investigate whether the blockade of AKR1C1 with a pharmacological inhibitor could restore sensitivity to gefitinib. The CCK8 data revealed that the combination of gefitinib and ALA had greater antiproliferative effects than gefitinib alone (Fig. S3A). Serial combination index values at each combination concentration indicated great synergism for the regimen of gefitinib plus ALA in both PC-9 and PC-9 GEF cells (Fig. 1G; Fig. S3B). In PC-9 and PC-9 OR cells, although the combination resulted in a greater proliferation inhibition rate than monotreatment did, the combination index values were all ∼0.82, indicating weak synergism for the regimen of osimertinib plus ALA (Fig. S3C–E). These data reveal that the therapeutic targeting of AKR1C1 with ALA has a great synergism with gefitinib but weakly synergizes with osimertinib in parental and resistant cells.

To further confirm the anti-tumor effects of the combination (gefitinib plus ALA), we measured the level of apoptosis (Fig. S3F). Consistent with the flow cytometry results, compared with monotreatment, 2-day cotreatment markedly increased the level of cleaved PARP protein, indicating increased apoptosis in the combination group (Fig. S3F; Fig. 1H). However, elevated cleaved PARP was not observed in the PC-9 OR cells, indicating that the combination of osimertinib and ALA had no augmented anti-tumor effects (Fig. 1I). Colony formation further demonstrated the long-term anti-tumor effects of the regimen (Fig. S3G, H). These newly revealed data provide evidence that enhanced apoptosis is induced by the combination of gefitinib and inhibitors targeting AKR1C1.

To explore the underlying mechanism, we focused on STAT3, a direct binding protein for AKR1C1 identified in our previous work.^2^ Elevated p-STAT3 levels were observed in PC-9 GEF cells (versus PC-9 cells), which were reversed by siAKR1C1 (Fig. 1J). After AKR1C1 was identified as a target of ALA in both PC-9 and PC-9 GEF cells by CETSA and ITDRF, we also observed the effects of ALA on p-STAT3 (Fig. S4C, D). Gefitinib increased p-STAT3 in PC-9 GEF cells in a concentration- and time-dependent manner (Fig. S4C and D), which was reversed by ALA (Fig. 1K). Consistent with the document,^5^ osimertinib treatment resulted in a slight decrease in p-STAT3, which may account for the weak synergistic effects of ALA and osimertinib (Fig. S4E). Next, the immunohistochemistry staining data revealed that AKR1C1 up-regulation, along with an increase in p-STAT3 levels in gefitinib-treated xenograft tumors, was strongly positively correlated (Fig. S4F, G). These data indicate that AKR1C1 confers resistance to gefitinib via STAT3, which is involved in the synergistic anti-tumor effects of the combination of ALA and gefitinib.

Compared with the monotherapies, the combination of ALA and gefitinib further impeded the growth of PC-9 GEF and PC-9 cell xenograft tumors, resulting in a significant reduction in tumor volume (Fig. S5A and D). Weight monitoring indicated that the combination did not cause significant body weight loss (Fig. S5B, E). Dissected PC-9 GEF cell xenograft tumors were photographed (Fig. S5C). Weight analysis of the PC-9 cell xenograft tumors also revealed the synergistic anti-tumor effects of gefitinib combined with ALA (Fig. S5F).

Finally, we evaluated the clinical potential of targeting AKR1C1 in the context of gefitinib resistance. For *EGFR*-mutated NSCLC patients receiving EGFR-TKI treatment, a significantly shorter disease-free survival was observed in patients with greater expression levels of *AKR1C1* (Fig. 1L). Furthermore, for Del19 EGFR-mutated NSCLC, OncoSG database analyses revealed a significantly shorter overall survival time for patients with greater expression levels of *AKR1C1* (Fig. S6A). For L858R *EGFR*-mutated NSCLC, we established a patient-derived xenograft (PDX) model (Fig. S6B). Gefitinib impeded PDX tumor growth, and the pharmacological targeting of AKR1C1 by ALA further significantly decreased tumor volume (Fig. 1M; Fig. S6C, D). ALA further decreased the tumor weight in this PDX model (Fig. S6E). AKR1C1 up-regulation, as well as the induction of nuclear STAT3, was observed in gefitinib-treated tumors and was abrogated by ALA treatment (Fig. 1N). The immunohistochemistry staining images revealed a positive correlation between AKR1C1 and nuclear STAT3 in PDX tumors (Fig. 1O). These data highlight the promising clinical potential of the pharmacological targeting of AKR1C1 in gefitinib treatment.

Collectively, the major findings of this work are as follows: ⅰ) AKR1C1 confers resistance to gefitinib in *EGFR*-mutated NSCLC; ⅱ) the pharmacological targeting of AKR1C1 with ALA has a strong synergism with gefitinib; and ⅲ) STAT3 may dictate AKR1C1-mediated gefitinib resistance (Fig. S7). These findings underscore the important role of AKR1C1 in gefitinib resistance and present a new exciting strategy for targeting AKR1C1 to enhance the anti-tumor effects of gefitinib monotherapy, opening a new avenue for delaying gefitinib resistance in advanced NSCLC.

## CRediT authorship contribution statement

**Linlin Chang:** Conceptualization, Data curation, Formal analysis, Funding acquisition, Investigation, Methodology, Writing – original draft. **Shuzhen Wei:** Data curation, Formal analysis, Methodology, Writing – original draft, Validation. **Xiaotian Qi:** Data curation, Writing – original draft, Funding acquisition. **Yang Gao:** Methodology. **Jinhua Chen:** Methodology. **Yu Cui:** Formal analysis, Methodology. **Pengxing He:** Conceptualization, Formal analysis, Project administration, Supervision, Writing – review & editing. **Wenzhou Zhang:** Project administration, Supervision, Writing – review & editing.

## Ethics declaration

Animal studies were approved by the Animal Research Committee at the Laboratory Animal Center of Henan Province (Henan, China) with ethical approval number ZZU-LAC20220729 [10,31,32], and comply with the animal Care and use rules of Zhengzhou University. The PDX studies were approved by the Medical Ethics Review Board at the Zhengzhou University Affiliated Cancer Hospital with ethical approval number 2022-KY-0157-001.

## Funding

This work was supported by the 10.13039/501100001809National Natural Science Foundation of China (No. 82102741 to L. Chang), Young Talent Support Project of Henan Province, China (No. 2024HYTP042 to L. Chang), 10.13039/501100011447Henan Provincial Science and Technology Research Project (China) (No. 242102310061 to L. Chang), Shandong Provincial Natural Science Foundation (China) (No. ZR2021QH100 to X. Qi), and China Zhongguancun Precision Medicine Science and Technology Foundation (No. ZGC-YXKY-19 to L. Chang).

## Conflict of interests

The authors declared no conflict of interests.
