# Supplementary material for: The targeting of AKR1C1 synergizes with gefitinib via the STAT3 signaling pathway in EGFR-mutated NSCLC
Source: Genes Dis. 2025 Apr 10;12(6):101633. doi: 10.1016/j.gendis.2025.101633 (PMC12272410; doi:10.1016/j.gendis.2025.101633)
Supplement: Multimedia component 1 [file mmc1.docx]

**The targeting of AKR1C1 synergizes with gefitinib via the STAT3 signalling pathway in *EGFR*-mutated NSCLC**

Linlin Chang^a^, Shuzhen Wei^b^, Xiaotian Qi^c^, Yang Gao^a^, Jinhua Chen^a^, Yu Cui^d^, Pengxing He^b*^, Wenzhou Zhang^a*^

*a. Department of Pharmacy, The Affiliated Cancer Hospital of Zhengzhou University & Henan Cancer Hospital; Henan Engineering Research Center for Tumour Precision Medicine and Comprehensive Evaluation, Henan Cancer Hospital; Henan Provincial Key Laboratory of Anticancer Drug Research, Henan Cancer Hospital, Zhengzhou 450008, China*

*b. School of Pharmaceutical Sciences, Zhengzhou University, Zhengzhou 450001, China*

*c. Key Laboratory of Immune Microenvironment and Inflammatory Disease Research in universities of shandong province，School of Basic Medical Sciences, Shandong Second Medical University, Weifang, Shandong province, 261053, China*

*d. Department of Hematopathy, Henan Institute of Hematology, The Affiliated Cancer Hospital of Zhengzhou University & Henan Cancer Hospital, Zhengzhou, 450008, Henan, China.*

*Corresponding authors.

*E-mail addresses:* [hepengxing@zzu.edu.cn](mailto:hepengxing@zzu.edu.cn) (P. He), [zlyyzhangwenzhou0551@zzu.edu.cn](mailto:zlyyzhangwenzhou0551@zzu.edu.cn) (W. Zhang).

**Materials and methods**

**Reagents**

Gefitinib (Gefi, Product No. T1181, CAS No. 184475-35-2), osimertinib (Osi, Product No. T2490, CAS No. 1421373-65-0) and alantolactone (ALA, Product No. T2896, CAS No. 546-43-0) were both purchased from TargetMol (MA, USA). Chemicals used *in vitro* were dissolved in DMSO and stored at – 80 ◦C.

**Cell lines**

The human non-small cell lung cancer cell lines PC-9 (the parental cell line) and HCC827 were obtained from the Shanghai Institutes for Biological Sciences (Cell Bank of the Chinese Academy of Sciences, Shanghai, China). PC-9 was cultured in gradually increased sublethal doses of gefitinib or osimertinib over generations to establish PC-9 GEF (the cell line resistant to gefitinib) or PC-9 OR (the cell line resistant to osimertinib). HCC827 and PC-9 are non-small cell lung cancer (NSCLC) cell lines that harbor the EGFR exon 19 deletion (Del19) mutation, which is one of the gefitinib-sensitive EGFR mutations. Another gefitinib-sensitive EGFR-activating mutation is the L858R point mutation in exon 21. T0, T2, and T4 cell lines were enriched according to our previous work.^1^ Cells were all maintained in RPMI 1640 plus 10% fetal bovine serum (FBS, SX1500, SORFA). The cell culture incubator was maintained at 37 °C with a humidity level, containing a mixture of 20% oxygen (O2) and 5% carbon dioxide (CO2).

**Microarray analysis**

Cells were plated in a 6-well plate. 36 hours later, the cells were lysed in TRIzol reagent (15596026, Invitrogen, Carlsbad, CA, USA) and analyzed using microarray analysis (SurePrint G3 Human Gene Expression 8× 60K v2, Agilent Array®). The gene expression profiles were analyzed using TreeView and Cluster 3.0 software.

**Western blotting**

The primary antibodies used in this work were as follows: pY705-STAT3 (1:3000, #9145S), PARP (1:2000, #9532S), and STAT3 (1:2000, #9139S), all purchased from Cell Signaling Technology (CST, Danvers, MA, USA). AKR1C1 (1:5000, #GTX105620) was obtained from GeneTex (Irvine, CA, USA). β-Actin (1:2000, #db10001) was sourced from DiagBio (Hangzhou, Zhejiang, China).

**CCK8 (Cell Counting Kit-8) assay**

Cells (3-7 × 10^3^ cells/well) were seeded onto a 96-well plate and cultured overnight. Afterward, the cells were treated with the indicated concentrations of chemicals for three days. Discard the cell culture medium and add sterile PBS (90 μL/well) to the 96-well plate, followed by the CCK8 assay. CCK8 was purchased from TargetMol (#C0005, MA, USA).

**Colony formation**

Cells (0.5 × 10^3^ or 1 × 10^3^ cells/well) were seeded into a 12-well plate. The next day, the cells were treated with the indicated chemicals. Every three days, fresh medium was replenished. Cells were cultured for about 10-15 days and then fixed with trichloroacetic acid, followed by staining with Sulforhodamine B (SRB, S1402-25G, CAS No. 3520-42-1). SRB was purchased from Sigma-Aldrich Trading Co., Ltd. (#Shanghai, China).

**siRNA and plasmid transfection**

Cells were plated onto a 6-well plate for transfection (2 × 10^5^ cells/well for transient knockdown and 3 × 10^5^ cells/well for transient overexpression) according to the manufacturer’s instructions for Oligofectamine (#12252-011, Invitrogen, CA, USA) and jetPRIME® (#101000046, Polyplus, NY, USA). All siRNA was obtained from GenePharma Co. Ltd (Shanghai, China). For AKR1C1 knockdown, cells received siRNA treatment for twice. The human siRNA target sequences are presented below.

siAKR1C1#1: 5’-AAGCTTTAGAGGCCACCAAAT-3’.

siAKR1C1#2: 5’-GACACAGAGGATGGCTCTATG-3’.

**Lentivirus delivery system**

Human GV493-Scramble (hU6-MCS-CBh-gcGFP-IRES-puromycin), GV493-shAKR1C1#1, and GV493-shAKR1C1#2 lentiviruses were purchased from Jikai Gene Co., Ltd. (Shanghai, China). The target sequences of the lentiviruses are shown as follows:

shAKR1C1#1: 5’-ccggAAGCTTTAGAGGCCACCAAATctcgagATTTGGTGGC

CTCTAAAGCTTtttttg-3’. shAKR1C1#2: 5’- ccggGACACAGAGGATGGCTCT

ATGctcgagCATAGAGCCATCCTCTGTGTCtttttg -3’.

**Immunohistochemistry**

Immunohistochemistry (IHC) was conducted with primary antibodies against Ki67 (1:2000, #27309-1-AP), AKR1C1 (1:800, #GTX105620, GeneTex), pY705-STAT3 (1:200, #9145S, CST), and STAT3 (1:500, #9139S, CST). Ki67 was obtained from Proteintech (Wuhan, Hubei, China). For antigen retrieval, citrate/sodium citrate buffer (pH 6.0) was applied for AKR1C1 staining, and tris/EDTA buffer (pH 9.0) was applied for pY705-STAT3, STAT3, and Ki67 staining. IHC staining was quantified using Aipathwell software (Servicebio, Wuhan, China).

**Flow cytometry**

Cells (1 × 10^5^ cells/well) were plated in a 6-well plate overnight. The following day, the cells were treated with dimethyl sulfoxide (DMSO) or the indicated drugs for approximately two days. After digestion with trypsin, the cells were stained with propidium iodide (PI, C6031, Kermey) following the manufacturer’s instructions. The flow cytometry (BD FACSCalibur, BD Biosciences) was employed to detect the PI signal (1 × 10^4^ cells/sample). Flowjo V10 software was used to analyze the PI signal.

**Antitumour activity in vivo**

Animal studies were approved by the Animal Research Committee at the Laboratory Animal Center of Henan Province (Henan, China) with ethical approval number ZZU-LAC20220729[10], ZZU-LAC20220729[31], ZZU-LAC20220729[32]. These studies comply with the animal care and use rules of Zhengzhou University. About 4-week-old male BALB/c nude mice were purchased from Gempharmatech Co., Ltd. (Jiangsu, China) and housed in a standard pathogen-free (SPF) environment. A total of 6 × 10^6^ cells were injected subcutaneously to establish the human xenograft model *in* *vivo*. All the mice were randomly divided into four groups: the control group (1% sodium carboxymethyl cellulose or sterile normal saline), the gefitinib group (3 or 5 mg/kg, administered via oral gavage once daily), the alantolactone group (15 mg/kg, administered via tail vein injection every two days), and the combination group. In patient-derived xenograft (PDX) studies, NOD.Cg-*Prkdc*^scid^Il2rg^tm1Sug^/JicCrl (NOG) mice were purchased from Vital River Laboratory Animal Technology Co., Ltd. (Beijing, China). The PDX studies were approved by the Medical Ethics Review Board at the Zhengzhou University Affiliated Cancer Hospital, with ethical approval number 2022-KY-0157-001.

**Statistical analysis**

A two-tailed Student’s t-test was conducted to determine the significance between two groups. A two-way ANOVA was employed to evaluate intergroup differences. Statistical results are presented below: *, p < 0.05; **, p < 0.01; ***, and p < 0.001; n.s., p ≥ 0.05. The CORREL function in Excel software was used to calculate the correlation coefficient value (R). 0.5 < R < 1.0, a highly positive correlation.

**Fig. S1** AKR1C1 is involved in TKI resistance for NSCLC. **(A)** Heat map of expression levels of top genes in a previous TKI resistance model. **(B)** Hazard ratio for overall survival of lung cancer patients from the Kaplan-Meier plotter database. **(C)** Kaplan‒Meier plotter analysis of the overall survival of lung cancer patients with different AKR1C1 expression levels. **(D)** Immunohistochemical assays were conducted to evaluate the protein levels of AKR1C1 in PC-9 gefitinib-resistant xenograft tumours treated with the first-generation EGFR TKI (gefitinib). **(E)** AKR1C1 staining was quantified using Aipathwell software. **(F)** Western blotting revealed the expression levels of AKR1C1 in PC-9 parental, PC-9 GEF, and PC-9 OR cells.

**Fig. S2** AKR1C1 confers resistance to the first-generation EGFR TKI (gefitinib) in NSCLC. **(A)** Knockdown efficiency of siAKR1C1 was shown by Western blotting in PC-9 and PC-9 GEF. **(B)** Overexpression efficiency of AKR1C1 was shown by Western blotting in PC-9 shAKR1C1. **(C)** The overexpression efficiency of AKR1C1 was shown by Western blotting in HCC827. **(D)** A CCK8 assay was employed to evaluate the viability of HCC827 cells upon gefitinib treatment in the AKR1C1 overexpression group and the vector group. **(E)** Western blotting demonstrated the knockdown efficiency of siAKR1C1 in PC-9 and PC-9 OR cells. **(F)** Representative images from PC-9 GEF xenograft tumours are presented, obtained through an immunohistochemical assay.

**Fig. S3** The combination of gefitinib and the AKR1C1 inhibitor alantolactone (ALA) enhances apoptosis, resulting in augmented anticancer effects *in vitro*. **(A)** CCK8 assays were employed to evaluate the viabilities of PC-9 and PC-9 GEF cells upon treatment with gefitinib, the AKR1C1 inhibitor (ALA), or their combination. **(B)** CI values ± SD were presented at different concentrations of drugs. **(C)** Viabilities of PC-9 and PC-9 OR were evaluated by the CCK8 assay with osimertinib treatments, AKR1C1 inhibitor (ALA) treatments, or their combination. **(D)** CI (combination index) values (mean ± SD) were presented at different concentrations of osimertinib and ALA. **(E)** CI values for each combination were shown in different circles (black circles for PC-9, white circle for PC-9 OR). **(F)** The Annexin V-FITC/PI staining assay was conducted to assess the apoptotic cells in PC-9 and PC-9 GEF cells treated with ALA, gefitinib, or their combination. PC-9 cells were treated with 2 μM ALA, 2.5 μM gefitinib, or their combination for three days. PC-9 GEF cells were treated with 5.0 μM ALA, 5.0 μM gefitinib, or their combination for three days. **(G)** (PC-9) and **(H)** (PC-9 GEF), cloning assays were employed to study the viabilities of cells under different treatments. PC-9 cells were treated with 1.0 μM ALA, 0.625 μM gefitinib, or their combination in 12-well plates for 3 days. After this period, the cells were replenished with fresh medium and cultured for another 7 days, followed by SRB staining. Similarly, PC-9 GEF cells were treated with 1.0 μM ALA, 7.5 μM gefitinib, or their combination in 12-well plates for 3 days. They were then replenished with fresh medium and cultured for another 7 days, followed by SRB staining.

**Fig. S4** The AKR1C1-STAT3 signalling pathway contributes to gefitinib resistance. **(A)** The Cellular Thermal Shift Assay (CETSA) was conducted to determine the thermal stabilization of AKR1C1 targeted by ALA. **(B)** The isothermal dose-response fingerprinting (ITDRF) experiment was performed at 53°C to show dose-dependent stabilization of AKR1C1 by ALA. **(C)** The p-STAT3 level was monitored by Western blotting after treatment with escalating concentrations of gefitinib for 24 hours in PC-9 GEF cells. **(D)** Treated with 5 μM gefitinib, PC-9 GEF cells were cultured for different durations as indicated, followed by evaluation of p-STAT3. **(E)** The p-STAT3 level was monitored by Western blotting after treatment with escalating concentrations of osimertinib for 24 hours in PC-9 OR cells. **(F)** Representative images display IHC staining for AKR1C1 and p-STAT3. Scale bar: 200 μm. **(G)** AKR1C1 correlates with p-STAT3 in xenograft tumours. Each plot represents the expression of AKR1C1 and p-STAT3 in a separate mouse tumour. p-STAT3 and AKR1C1 staining were quantified using Aipathwell software. The correlation coefficient value (R) was calculated using the CORREL function in Excel. 0.5 < R <1.0, a highly positive correlation.

**Fig. S5** AKR1C1 inhibitor sensitizes the antitumour effect of gefitinib in PC-9 and PC-9 GEF xenograft mice. The tumour volume is presented as the mean ± SD for PC-9 GEF **(A)** or PC-9 **(D)**. Each group contains five nude mice. Body weight is monitored, as shown for PC-9 GEF **(B)** and for PC-9 **(E)**. **(C)** Tumours were dissected and photographed for PC-9 GEF xenograft mice. **(F)** Dissected tumours were weighed and reported as mean ± SD for PC-9 xenograft mice.


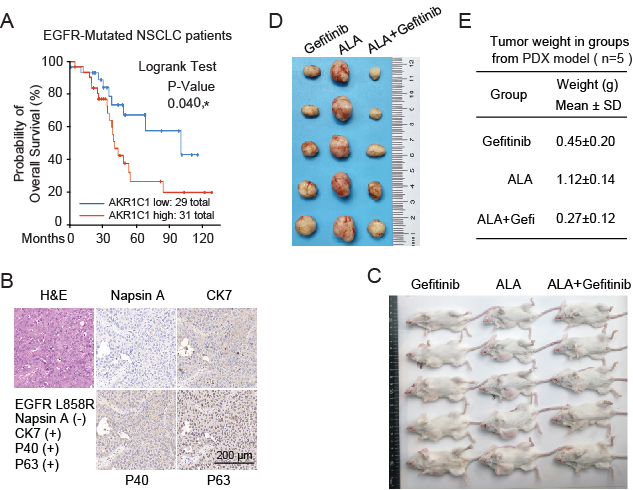
**Fig. S6** Promising potential of targeting AKR1C1 in NSCLC patients undergoing gefitinib treatment. **(A)** Survival analysis was performed on NSCLC patients with Del19 mutations in *EGFR* using the OncoSG database. **(B)** The pathological phenotypes of the PDX model are displayed. The tumour-bearing mice **(C)** and harvested tumour tissues **(D)** were photographed. **(E)** Tumour weight is recorded as mean ± SD.


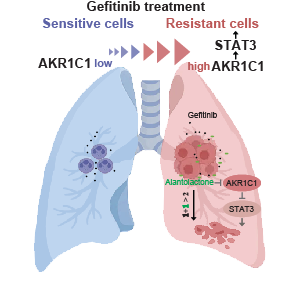


**Fig. S7** The targeting of AKR1C1 synergizes with gefitinib via the STAT3 signalling pathway in *EGFR*-mutated NSCLC.
